# Correlation Between the Prevalence of Myasthenia Gravis and the Frequency of Class II Human Leucocyte Antigen Alleles in Various Geographical Locations Around the World

**DOI:** 10.7759/cureus.69791

**Published:** 2024-09-20

**Authors:** Mathew Kurian, Nikhil Khera

**Affiliations:** 1 General Internal Medicine, University of Leicester Hospitals, Leicester, GBR; 2 General Practice, University of Leicester Hospitals, Leicester, GBR

**Keywords:** autoimmune disorders, prevalence studies, hla class ii genotype, genetic screening, global epidemiology, human leukocyte antigen (hla), myasthenia gravis (mg)

## Abstract

Myasthenia gravis (MG) is an autoimmune condition characterised by muscle weakness due to antibodies produced against post-synaptic receptors. The impact of MG can be significant, especially with an ageing population. Human leukocyte antigens (HLA) are polymorphic genes associated with autoimmune conditions. Establishing the HLA alleles associated with MG may aid in the diagnosis, screening and early management of individuals at risk of MG.

This research aims to establish the class II HLA alleles associated with the prevalence of MG in various regions of the world and identify the alleles that could predispose to the condition.

A Preferred Reporting Items for Systematic Reviews and Meta-Analyses (PRISMA) flow chart and various databases including, Scopus and PubMed as well as other sources were used to find appropriate papers on HLA class II alleles associated with MG and the prevalence of MG in various countries. The frequency of selected HLA alleles in selected regions were obtained from the website, allelefrequencies.net. From this, a correlation coefficient and p-value were calculated to investigate whether the frequency of MG and the prevalence of HLA alleles had a significant association.

The results highlighted two HLA alleles, DRB1*04:04 and DRB1*03, to have a significant positive association with the prevalence of MG. The frequency of the alleles showed regional variation, with European countries, particularly Northern Europe, exhibiting the highest frequencies.

A significant positive correlation between HLA-DRB1*04:04 and DRB1*03 showed with the prevalence of MG, highlighting these alleles as a possible cause of the disease. Screening for these alleles, particularly in Northern Europe, may help identify individuals susceptible to MG.

## Introduction and background

Aim

This research aims to investigate the relationship between the prevalence of acetyl-choline-induced myasthenia gravis (MG) in various geographical locations and the frequency of certain class II human leukocyte antigens (HLA) alleles in those same locations. The correlation between these two variables can be established and, if significant, can help identify the HLA alleles that may predispose to the development of MG.

Human leucocyte antigens

The term autoimmune disease refers to conditions whereby the body’s own immune system attacks the body, resulting in physiological damage. The variations in certain HLA alleles are known to predispose to some of these diseases. 

HLA are the most polymorphic genes within the human genome [1]. Existing on chromosome 6p21, they encode for the major histocompatibility complex (MHC), proteins responsible for the presentation of antigens to T-cells and hence the activation of T-cell-mediated immunity. There are three main classifications of HLA genes.

HLA class I: Exhibited in all nucleated cells, it expresses intracellular antigens onto the MHC class 1 proteins via the endogenous process to be recognised by CD8+ cytotoxic T-cells.

HLA class II: Exhibited in antigen-presenting cells (APCs), it presents extracellular antigens onto the MHC class 2 protein via the endogenous process to be recognised by CD4+ helper T-cells.

HLA class III: This is involved in inflammation and encodes for the formation of complements.

Polymorphism and polygenetic aspects of HLA alleles

The polymorphism of HLA is the result of the wide variety of antigens in the environment that humans are exposed to. The selection of mutated HLA genes that can respond to these antigens and hence mediate a T-cell response has led to the polygenetic nature of HLA, especially in the class I and II regions [2]. Moreover, there are various alleles and sub-alleles for each gene that adds to the diversity of HLA. The three main genes of HLA class I include: HLA-A, HLA-B and HLA-C3 [3].

Of the three HLA regions, class II HLA exhibits the greatest polymorphism and gene density [1]. Genes most frequently associated with class II include HLA-DPB, HLA-DQB and HLA-DRB, with HLA-DRB being the most frequent. There has also been a stronger association of HLA class II alleles with several autoimmune conditions compared to other classes of HLA [1].

Certain HLA alleles are found more commonly in certain groups and populations. It is therefore possible to study the correlation between specific HLA alleles and the development of certain autoimmune conditions. This review will investigate the correlation between certain HLA alleles and their association with myasthenia gravis.

Myasthenia gravis

Myasthenia gravis (MG) is an autoimmune condition belonging to a group of conditions known as synaptopathies, a group of rare neuromuscular junction (NMJ) disorders, characterised by the fatigability of voluntary muscles [4,5]. *Pathogenesis* The pathogenesis of MG is determined by the types of pathogenic antibodies produced and therefore the respective post-synaptic receptors with which they form a complex. Three antibodies have been identified: acetylcholine receptor antibodies (Acetyl-MG), muscle-specific kinase (MuSK) and the low-density lipoprotein-related receptor protein 4 (LRP4), with the most common form being Acetyl-MG [5]. This review will focus on this form of MG in identifying its correlation with certain HLA alleles. As most antibody responses are dependent upon CD4+ T cells, whose function is restricted by Class II HLA molecules, Class II HLA alleles are the most relevant.

*Epidemiology* Myasthenia gravis is a relatively rare autoimmune condition. However, population-based studies from the past 50 years have highlighted a clear trend towards an increase in the prevalence of MG, which is related to ageing populations in various developed countries [6]. There is also a bimodal distribution of MG related to sex and age. Females have a 2:1 prevalence of MG as compared to males aged 20-30, whereas in older ages above 50, males are more commonly affected than females [7]. Therefore as the population ages, the incidence of MG in men is now higher than that for females [7]. The increasing trend could also be explained by the hygiene hypothesis, which proposes that less exposure to pathogens at a young age in developed countries leads to increased incidence of allergies and autoimmune diseases.

*Sub-types of MG* MG can be classified into various categories. Clinically, it can be subdivided into early-onset, with the onset of symptoms before <50 years, and late-onset, defined as >50 years, which is more common in males [8]. As thymic disorders and their association with MG are well-established, thymoma-associated MG is also considered a sub-type of MG [8]. Ocular-onset MG represents a sub-group characterised by the weakness of ocular muscles. A final sub-group is based on MusK and LRP antibodies, as well as antibody-negative generalized MG [8].

*Clinical Features*****

Initial symptoms: The symptoms of MG may be subtle and varied. Most patients complain of specific muscle weakness and fatigability that affects a specific group of muscles [7]. As fast-twitch muscle fibres are often involved, approximately two-thirds of cases involve the extrinsic ocular muscles resulting in conditions such as binocular diplopia and asymmetrical ptosis, which may resolve by covering one eye [7-9]. In 1/6 of patients, initial symptoms include difficulty in eating, swallowing or talking due to a weakness of the oropharyngeal muscle and jaw closure muscles [7,9]. Only 10% of patients initially present with limb weakness [7,9]. MG typically has a diurnal variation with symptoms manageable in the morning and getting worse during the day after continuous use of the affected muscles [7]. Moreover, symptoms are often exacerbated by exercise and an increase in body temperature [7,9].

Progression of symptoms: Most patients will present with ocular symptoms within two years [7]. However, the progression of the disease affecting other muscles varies among patients. In 10-40% of cases, weakness is restricted to the ocular muscles [7]. In the remaining, there is ongoing involvement of the oropharyngeal and limb muscles, resulting in the previously mentioned complications [7]. Although the disease may be localised in some patients, others may progress to generalised weakness and fatigue [10]. In severe cases, this may extend to muscle weakness of respiratory muscles and a subsequent need for mechanical ventilation [10]. It is also estimated that after 15-20 years, the weakness of affected muscles becomes permanent and those most severely affected may start to atrophy [7].

Reasons for the research

The management of MG is individualised according to the patient’s disease severity, sex, age and other factors [11]. Treatment options, including anti-acetylcholinesterase agents, corticosteroids, immunosuppression drugs, thymectomy and plasmapheresis, have been shown to improve symptoms and even allow patients to go into remission [11]. Although there is proven beneficence from the mentioned treatments, there are no current cures for MG. Muscle weaknesses leading to disability, decreased quality of life and, in severe cases, hospitalisation are still pertinent.

Moreover, with epidemiological evidence revealing an increase in MG prevalence, the need to understand the disease and investigate potential management strategies becomes even more necessary. Although much has been done to provide a brighter prognosis, research into the correlation between HLA alleles and MG may provide a better understanding of the disease and potentiate a screening tool for early identification and management.

Ethics

As this search involves data interpretation from published papers and articles, no ethical considerations were involved in this research. Healthy subjects' tissue typed as potential bone marrow donors was consented to by individual laboratories contributing data to the allelefrequencies.net website.

## Review

Methods

**Figure 1:**
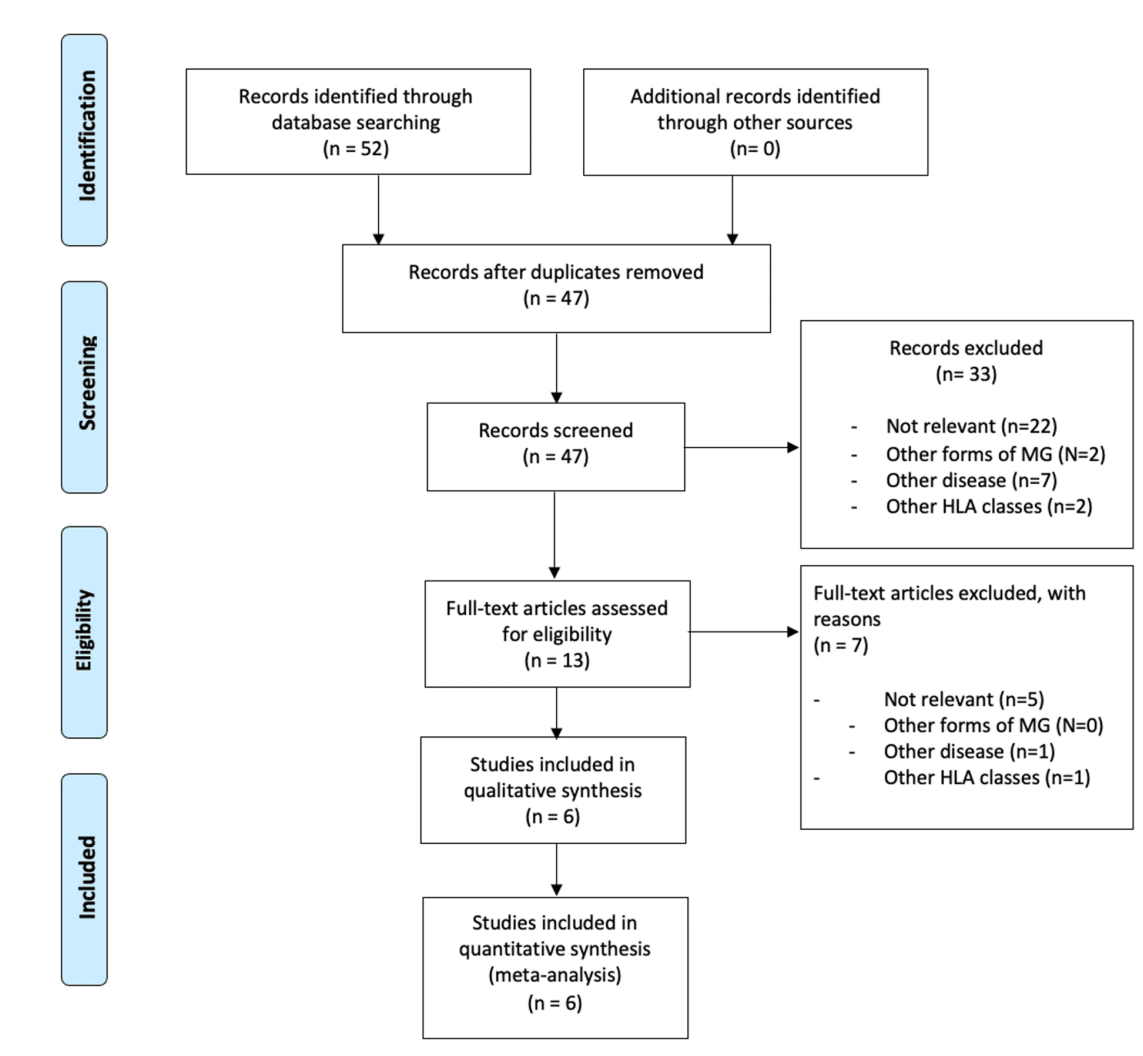
PRISMA flow diagram 1 highlights the key step in identifying papers associating HLA alleles with MG Adapted from [12] PRISMA: Preferred Reporting Items for Systematic Reviews and Meta-Analyses, HLA: human leukocyte antigen, MG: myasthenia gravis

Initially, research was conducted to identify which HLA alleles are thought to predispose to MG. Papers investigating the HLA alleles were selected using the Preferred Reporting Items for Systematic Reviews and Meta-Analyses (PRISMA) flow diagram as highlighted below (Figure 1).

Records Identified Through Database Searching

Database search engines used for the initial identification of records include Scopus and PubMed (Table 1). Once the initial papers were identified and duplications removed, the title and abstract of the remaining papers were screened for eligibility (Figure 1).

**Table 1:** The databases used, key search terms implemented, limitations applied and number of results obtained from each database

		Key search terms used and results obtained		
Limitation applied to each database	Databases	“Myasthenia gravis”	"Human leukocyte antigen "	“Myasthenia Gravis” AND "Human leukocyte antigen "
Documentation - article and review, human species, Language: English, Date: 01/01/1990-2017	Scopus	11420	14433	46
	PubMed	9658	1787	5

The inclusion criteria included papers involving acetyl-MG, as well as all subtypes of acetyl-MG, as this accounts for the majority of MG cases (Table 2). HLA class II alleles were studied as opposed to other HLA classes due to their increased association with autoimmune disease (Table 2).

**Table 2:** Exclusion and inclusion criteria (and reasons) employed for the initial screening of records to identify papers associating MG with certain HLA alleles MG: myasthenia gravis, HLA: human leukocyte antigen

Inclusion criteria	Exclusion criteria	Rationale
Acetyl-MG	- Other forms of MG, i.e. MusK and LRP4-induced MG, - Other autoimmune conditions	Acetyl-MG accounts for 85% of MG cases
All sub-types of acetyl-MG		All sub-types of MG can be associated with class II HLA-alleles
Class 2 HLA including alleles and haplotypes of class 2	Class 1 HLA	Class 2 HLA is more associated with autoimmune conditions than other HLA classes

**Figure 2:**
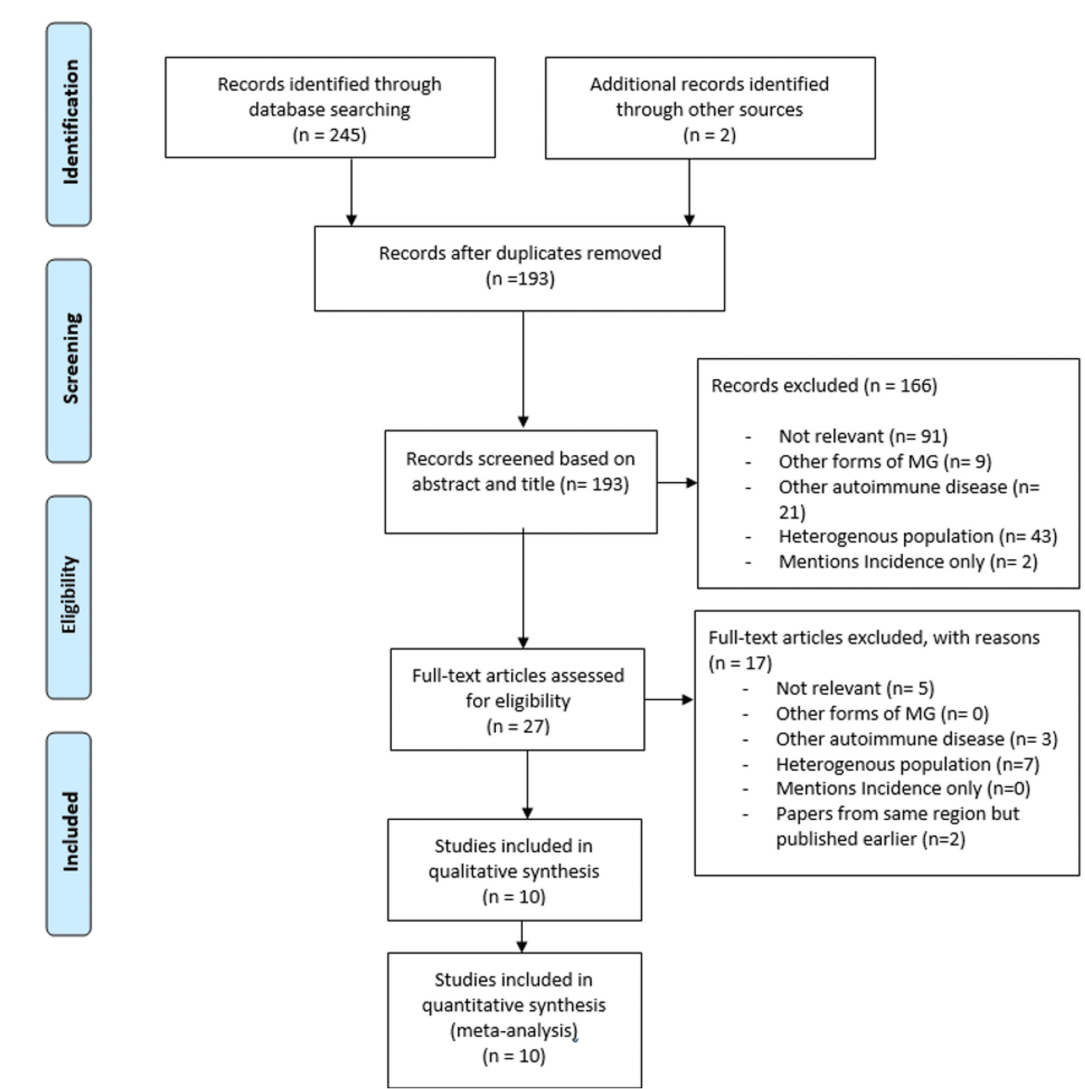
PRISMA flow diagram 2 highlights the key steps in identifying papers investigating the prevalence of MG in various regions Adapted from: [12] PRISMA: Preferred Reporting Items for Systematic Reviews and Meta-Analyses, MG: myasthenia gravis

Once the HLA antigens associated with MG were established, research was conducted to find the prevalence of MG in various regions (Figure 2).

Databases Searched

The database search engines used for the initial identification of records include Scopus and PubMed (Table 3).

**Table 3:** The database involved, key search terms used and results obtained when investigating the prevalence of MG in various regions MG: myasthenia gravis

		Key search terms used and results obtained from each database			
Limitations applied to each database	Database	“Myasthenia Gravis”	“Epidemiology”	“Myasthenia gravis” AND “Epidemiology”	“Myasthenia gravis” AND “Epidemiology” AND “Prevalence”
Articles and reviews, human species, date: 01/01/1990-2017	Scopus	12,557	316,487	193	82
	PubMed	9546	1,342,578	613	163

Records Screened

The exclusion and inclusion criteria for selecting papers for further full-text eligibility screening are highlighted in Table 4. Areas with a homogenous demographic allow for a more reliable correlation to be assessed (Table 4). 

**Table 4:** The exclusion and inclusion criteria (and reasons) employed when investigating the prevalence of MG in various regions MG: myasthenia gravis

Inclusion criteria	Exclusion criteria	Rationale
Acetyl-MG	Other forms of MG, i.e. MusK and LRP4-induced MG, Other autoimmune conditions	Acetyl-MG accounts for 85% of MG cases.
Prevalence or epidemiology of MG	Incidence of MG	The research is focusing on the prevalence of MG.
Populations with a homogenous demographic	Populations with a heterozygous demographic	A homozygous demographic allows a more reliable association to be found.

Having found the specific HLA alleles associated with MG, each allele was input into the website allelefrequencies.net, and the frequency of all the alleles in various regions was obtained. Based on the prevalence of MG, screening was done to find the frequency of the selected HLA alleles in respective regions. If there is more than one frequency data for an HLA allele in one country, the region with the largest sample size was chosen. From this, a correlation between the frequency of the selected HLA alleles and the prevalence of MG in selected regions can be established and represented through a linear regression graph produced through Microsoft Excel (Microsoft Corporation, Redmond, WA, US). The strength of the correlation was calculated using the correlation coefficient (r) of the linear regression line. The larger the value of r, the stronger the association. To establish if the correlation was significant, the p-value was calculated, and a value of p< 0.05 was considered significant. From this, it can be established which HLA alleles are associated with MG and can therefore predispose to the condition.

Results

Research into identifying HLA alleles associated with MG established six papers identifying eight HLA class II alleles. Alleles of the HLA-DRB class II gene represented six out of the eight HLA alleles associated with MG and include HLA- DRB1*04:04, DRB1*01, DRB1*03, DRB1*04, DRB1*15:01 and DRB1*09 (Table 5). HLA-DQB1*02 and DQB1*03 of the class II gene represented the other alleles found. HLA-DRB1*03 and DRB1*09 were also identified to be associated with MG on two different papers (Table 5).

**Table 5:** Shows the papers obtained from the initial search to identify the HLA allele associated with MG HLA: human leukocyte antigen, MG: myasthenia gravis

Paper number	Title of paper	Author	Year of publication	HLA alleles identified to be associated with MG
1	Juvenile myasthenia gravis in Norway: HLA-DRB1*04:04 is positively associated with prepubertal onset [13]	Popperud, TH, Viken, MK, Kerty, E, et al.	2017	HLA-DRB1*04:04
2	HLA and age of onset in myasthenia gravis [14]	Santos, E, Bettencourt, A, da Silva, AM, et al.	2017	HLA- DRB1*01 HLA-DRB1*03
3	Association of HLA-DR/DQ polymorphism with myasthenia gravis in Tunisian patient [15]	Fekih-Mrissa, N, Klai, S, Zaouali, J, et al.	2013	HLA-DRB1*03 HLA-DQB1*02 HLA-DRB1*04 HLA-DQB1*03
4	Late onset myasthenia gravis is associated with HLA DRB1*15:01 in the Norwegian population [16]	Maniaol AH,Elsais A,Lorentzen AR,et al.	2012	HLA-DRB1*15:01
5	Association between HLA-DRB1 and myasthenia gravis in a northern Han Chinese population [17]	Xie, Y-C, Qu, Y, Sun, L, et al.	2011	HLA-DRB1*09
6	HLA class II and class I polymorphism in Venezuelan patients with myasthenia gravis [18]	Fernández-Mestre, MT, Vargas, V, et al.	2004	HLA-DRB1*09

Research into the prevalence of MG revealed patterns of prevalence depending on the region investigated. European countries exhibited the highest prevalence of MG (Table 6). Asian countries including Korea, Taiwan and Japan had a similar and relatively high prevalence of MG. American countries, including Mexico and Colombia, exhibited the greatest variety in MG prevalence (Table 6).

**Table 6:** The papers obtained to identify the prevalence of MG from various regions MG: myasthenia gravis

Regions	Paper No.	Title of paper	Author	Year of publication	Prevalence of MG/ 100,000
Korea	7	The epidemiology of myasthenia gravis in Korea [19]	Lee, H.S., Lee, H.S., Shin, H.Y., et al.	2016	13.0
Taiwan	8	Nationwide population-based epidemiological study of myasthenia gravis in Taiwan [20]	Lai, C.-H., Tseng, H.-F	2010	14.0
Japan	9	Characteristics of myasthenia gravis according to onset-age: Japanese nationwide survey [21]	Murai, H, Yamashita, N, Watanabe, M, et al.	2011	11.8
Denmark	10	Epidemiology of autoimmune diseases in Denmark [22].	Eaton, WW, Rose, NR, Kalaydjian, A	2007	17.85
Croatia	11	Incidence and prevalence of myasthenia gravis in the county of the coast and Gorski Kotar, Croatia, 1976 through 1996 [23].	Zivadinov, R, Jurjevic, A, Willheim, K, et al.	1998	9.9
Serbia	12	Epidemiological study of adult-onset myasthenia gravis in the area of Belgrade (Serbia) in the period 1979-2008 [24].	Lavrnic, D, Basta, I, Rakocevic-Stojanovic, V, et al.	2013	18.8
Norway	13	Myasthenia gravis epidemiology in a national cohort; combining multiple disease registries [25].	Andersen, JB, Heldal, AT, Engeland, A, et al.	2014	13.1
Mexico	14	Myasthenia gravis in adults of institutions pertaining to the Mexican public health system: an analysis of hospital discharges during 2010] [26].	Tolosa-Tort, P, Chiquete, E, Domínguez-Moreno, R, et al.	2015	11.1
Sweden	15	The autoimmune spectrum of myasthenia gravis: a Swedish population-based study [27].	Fang, F, Sveinsson, O, Thormars G, et al.	2002	24.8
Colombia	16	A first description of the Colombian national registry for rare diseases [28].	Mateus, HE, Pérez, AM, Mesa ML, et al.	2017	1.78

Main Regions Represented in the MG Prevalence Study

The main countries identified in this study can be summarised by their continental regions (Table 7).

**Table 7:** Countries represented in various regions based on research on MG prevalence MG: myasthenia gravis

Regions	Countries selected based on papers found
Asia	Korea, Taiwan, Japan
Northern Europe	Norway, Sweden, Denmark
Eastern Europe	Croatia, Serbia
Americas	Colombia, Mexico

Out of the eight HLA alleles found to be associated with MG through preliminary research, six alleles (HLA- DRB*04:04, DRB1*01, DRB1*03, DQB1*03, DRB1*04, DRB1*15:01) has a positive correlation with the prevalence of MG and 2 allele has a negative correlation (HLA-DRB1*09 and DQB1*03) (Table 8). Statistical analysis can be applied to determine the significance of the correlation and evaluate the impact the alleles have on MG prevalence. Although general population screening for these susceptible alleles may not be cost-effective, those at risk of developing MG e.g. family history of MG, can be screened to identify these alleles. This allows for the early recognition of the disease and makes earlier management feasible (Table 8). 

**Table 8:** Shows the correlation coefficient between each HLA allele and MG MG: myasthenia gravis, HLA: human leukocyte antigen

Strongest positive association with MG	HLA allele	Correlation coefficient (r)	P values
1	DRB1*04:04	+ 0.75	0.032
1	DRB1*03	+ 0.75	0.030
2	DQB1*02	+0.64	0.17
3	DRB1*01	+0.51	0.16
4	DRB1*:04	+0.48	0.19
5	DRB1*15:01	+ 0.17	0.67
6	DQB1*03	-0.14	0.85
7	DRB1*09	-0.23	0.59

**Figure 3:**
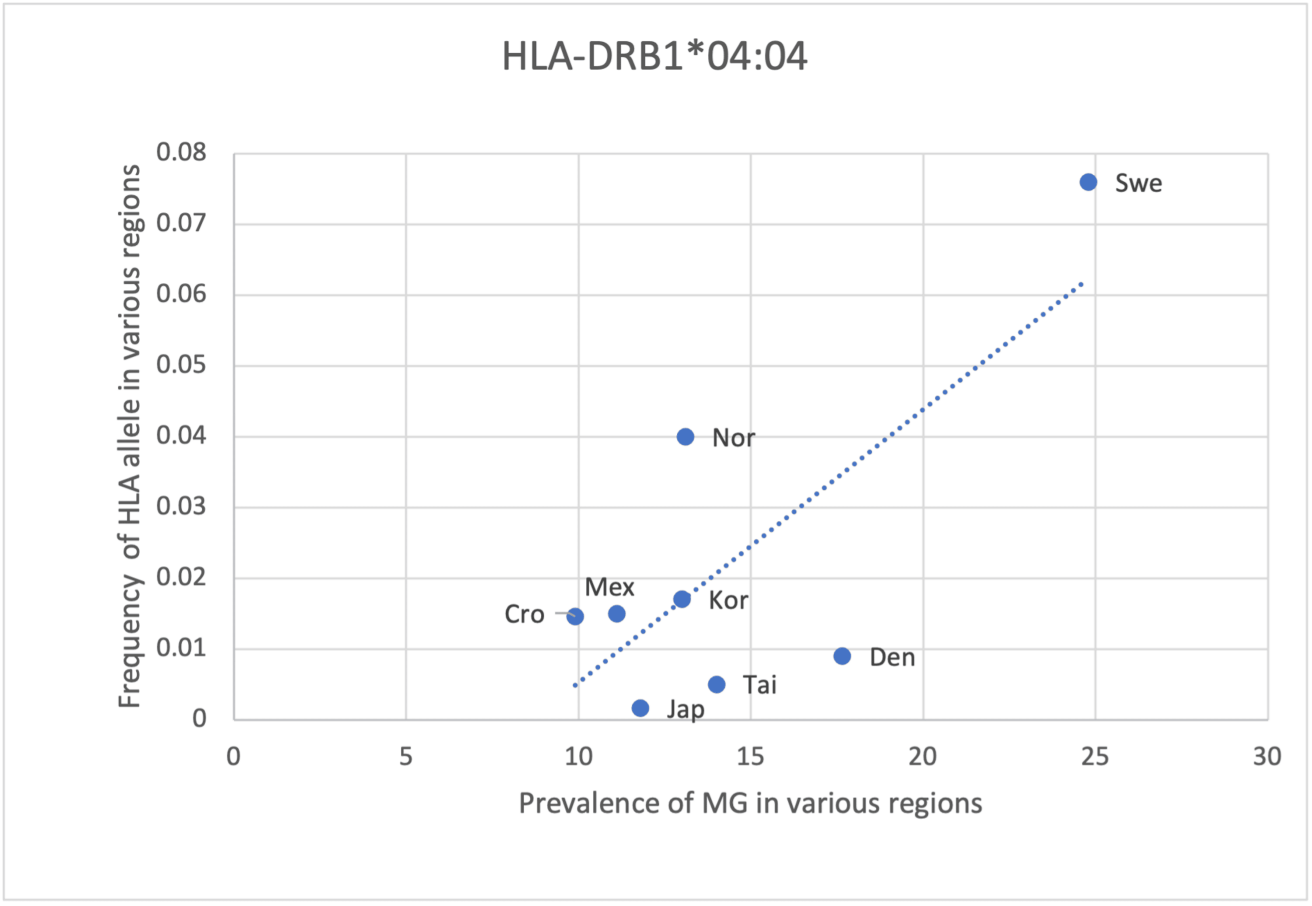
The correlation between the frequency of HLA-DRB1*04:04 in the various regions studied to the prevalence of MG in those same regions HLA: human leukocyte antigen, MG: myasthenia gravis

**Figure 4:**
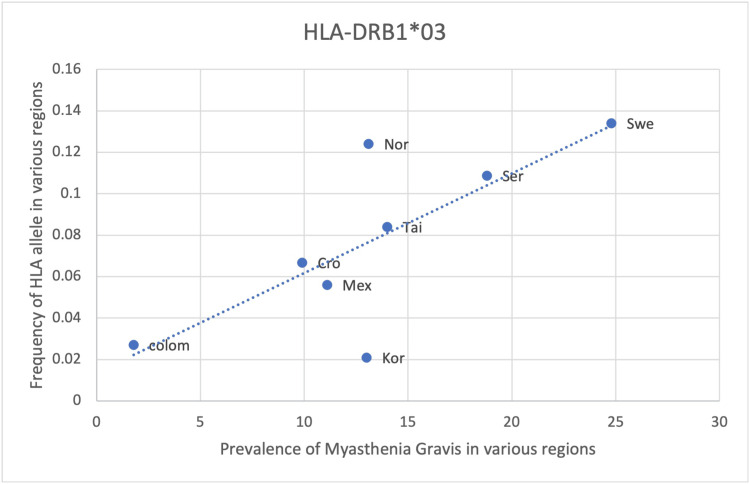
The correlation between the frequency of HLA-DRB1*03 in the various regions studied and the prevalence of MG in those same regions HLA: human leukocyte antigen, MG: myasthenia gravis

A statistically significant association (p <0.05) between the frequency of HLA alleles and MG prevalence was found in only two alleles: HLA-DRB1*04:04 and DRB1*03 (Table 8). A positive correlation with an r-value of 0.75 was observed in both alleles, suggesting DRB1*04:04 and DRB1*03 could predispose to the development of MG (Table 8). Regional differences also existed. Northern European countries, including Norway, Denmark and Sweden, as well as an Eastern European country represented by Serbia, exhibited the highest prevalence of these HLA alleles (Figure 3, Figure 4). In contrast, Asian (Taiwan, Korea and Japan) and American (Colombia and Mexico) countries had much lower prevalence (Figure 3, Figure 4). A possible explanation for this difference could be the result of the selection of these alleles to fight off infections or foreign antigens that are more prevalent in European countries. As there is a significantly positive correlation between HLA-DRB1*04:04 and DRB1*03 with MG; this would also suggest that the prevalence of MG in European countries is higher than in other regions, and this is seen from the prevalence data obtained for MG. Therefore, regional differences in HLA allele frequency suggest screening for these alleles would be best indicated in European countries, particularly Northern Europe, in identifying people at risk of MG (Table 8, Figures 3, Figure 4).

**Figure 5:**
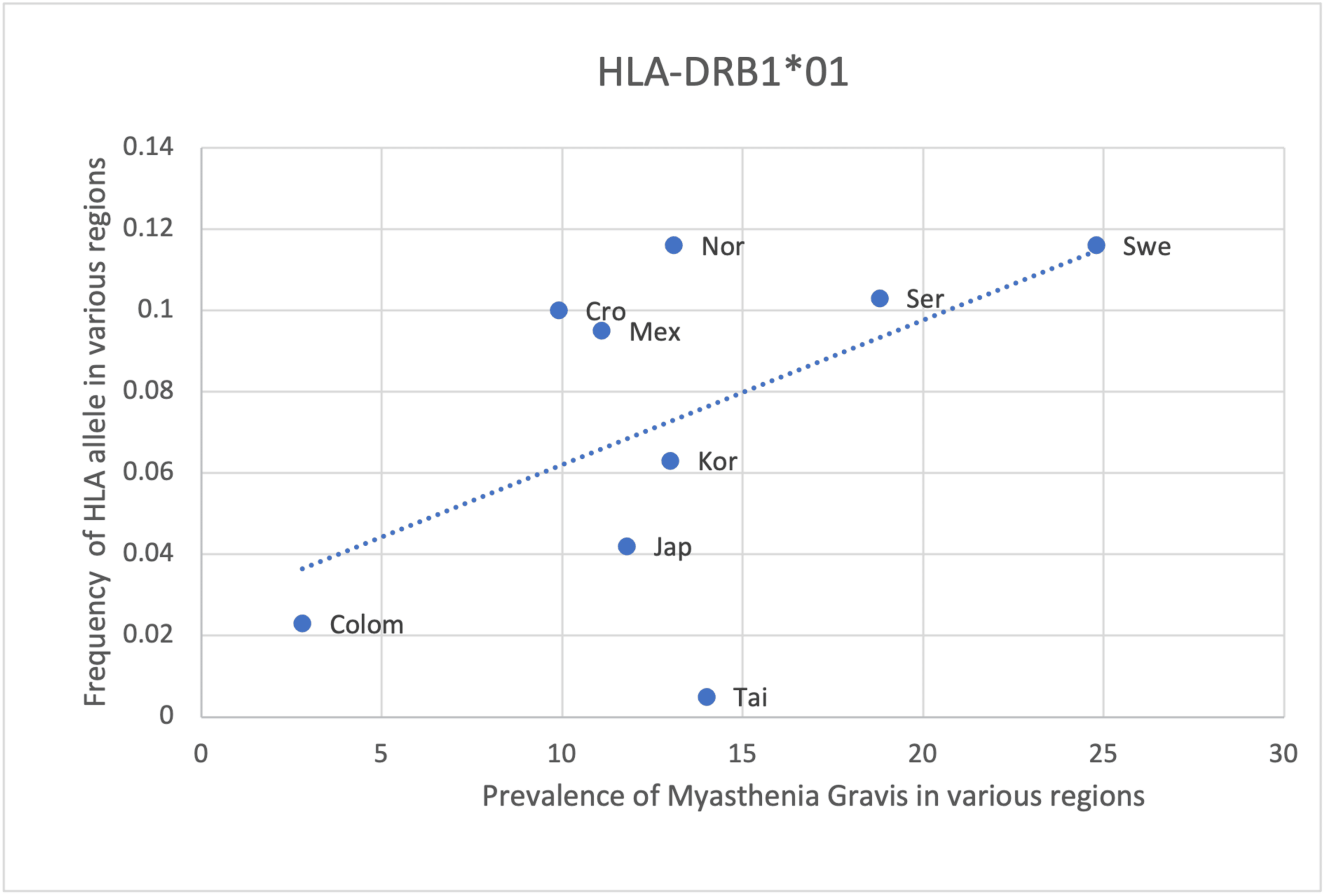
The correlation between the frequency of HLA-DRB1*01 in the various regions studied and the prevalence of MG in those same regions HLA: human leukocyte antigen, MG: myasthenia gravis

**Figure 6:**
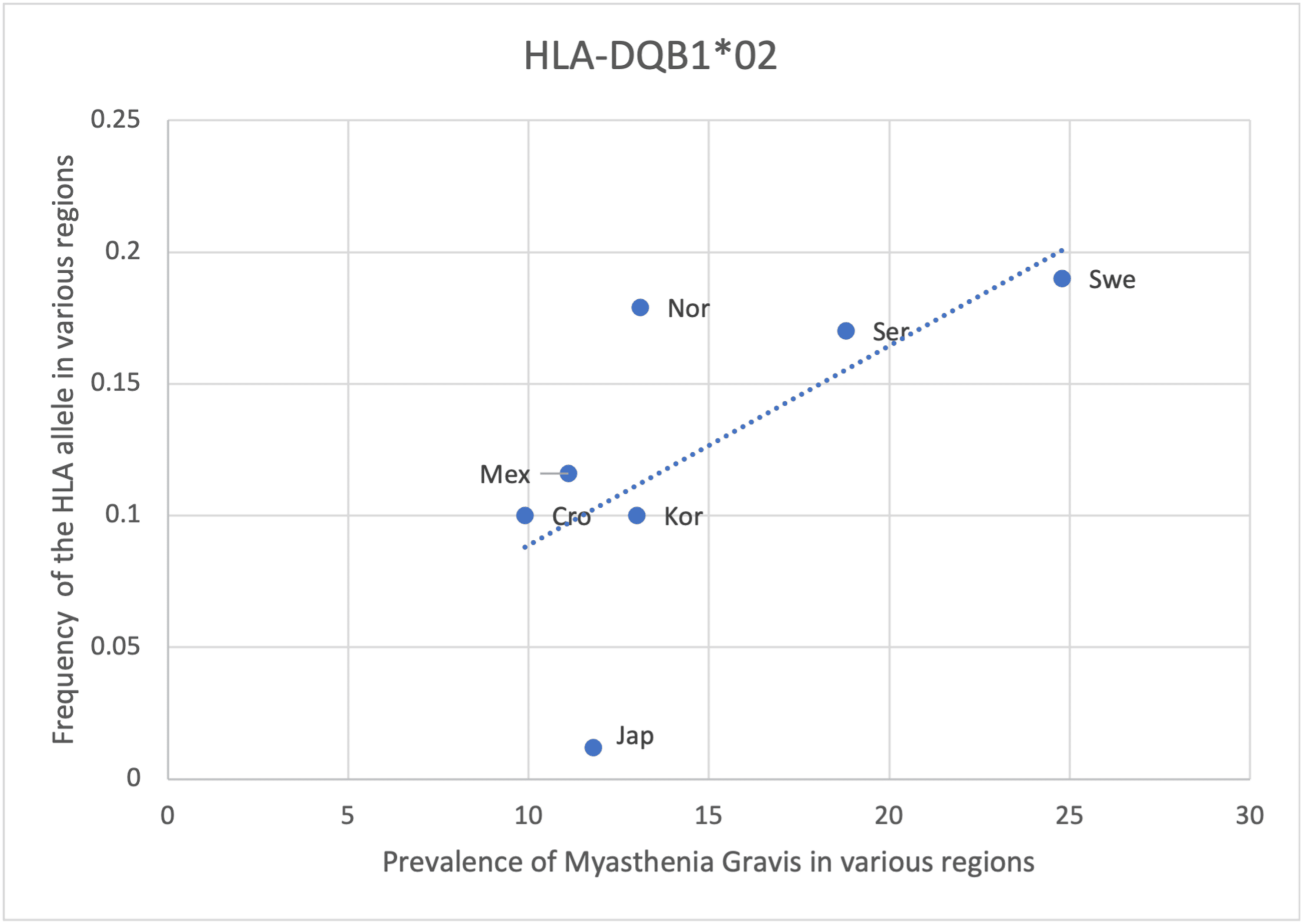
The correlation between the frequency of HLA-DQB1*02 in the various regions studied and the prevalence of MG in those same regions HLA: human leukocyte antigen, MG: myasthenia gravis

**Figure 7:**
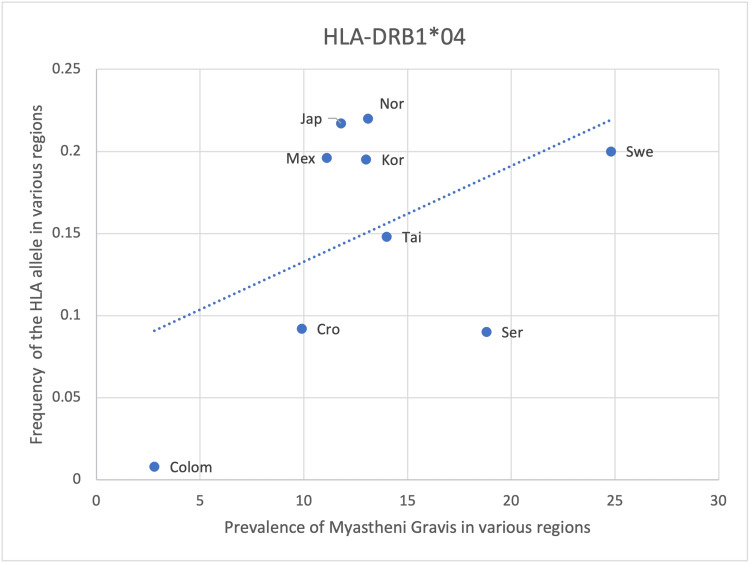
The correlation between the frequency of HLA-DRB1*04 in the various regions studied and the prevalence of MG in those same regions HLA: human leukocyte antigen, MG: myasthenia gravis

HLA- DRB1*01, DQB1*02 and DRB1*04 show a positive yet weaker correlation with the prevalence of MG with an r-value of 0.51, 0.64 and 0.48, respectively (Table 8, Figures 5-7). However, the statistical analysis revealed a p-value of >0.05 for all 3 alleles (Table 8). As such, the positive correlation observed is deemed insignificant and the respective HLA alleles cannot be said to be correlated with the prevalence of MG. The positive correlation observed could be due to random chance or an incidental finding. Another explanation could be that certain other factors may lead to an increase in the frequency of these alleles as well as an increase in the prevalence of MG, thus creating an impression of correlation between these two variables. These alternate causes may include infections, other genes and environmental factors (Table 8, Figures 5-7).

**Figure 8:**
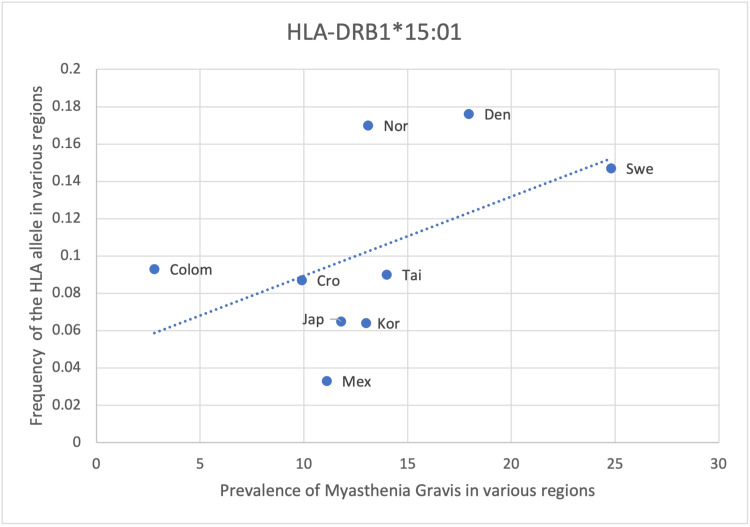
The correlation between the frequency of HLA-DRB1*15-01 in the various regions studied and the prevalence of MG in those same regions HLA: human leukocyte antigen, MG: myasthenia gravis

HLA-DRB1*15:01 showed the weakest positive correlation with MG with an r-value of 0.17 (Table 8, Figure 8). Regional variation also showed a higher prevalence of the allele in European countries as compared to Asian and American countries (Figure 8). However, statistical analysis revealed that the slight positive correlation observed was insignificant with a p-value >0.05 (Table 8). As mentioned above, the correlation could be an incidental finding or due to an association with other causative factors. Considering the correlation was weak, it can be assumed that HLA-DRB1*15:01 may not have any association with MG or factors pre-disposing to MG (Table 8, Figure 8).

**Figure 9:**
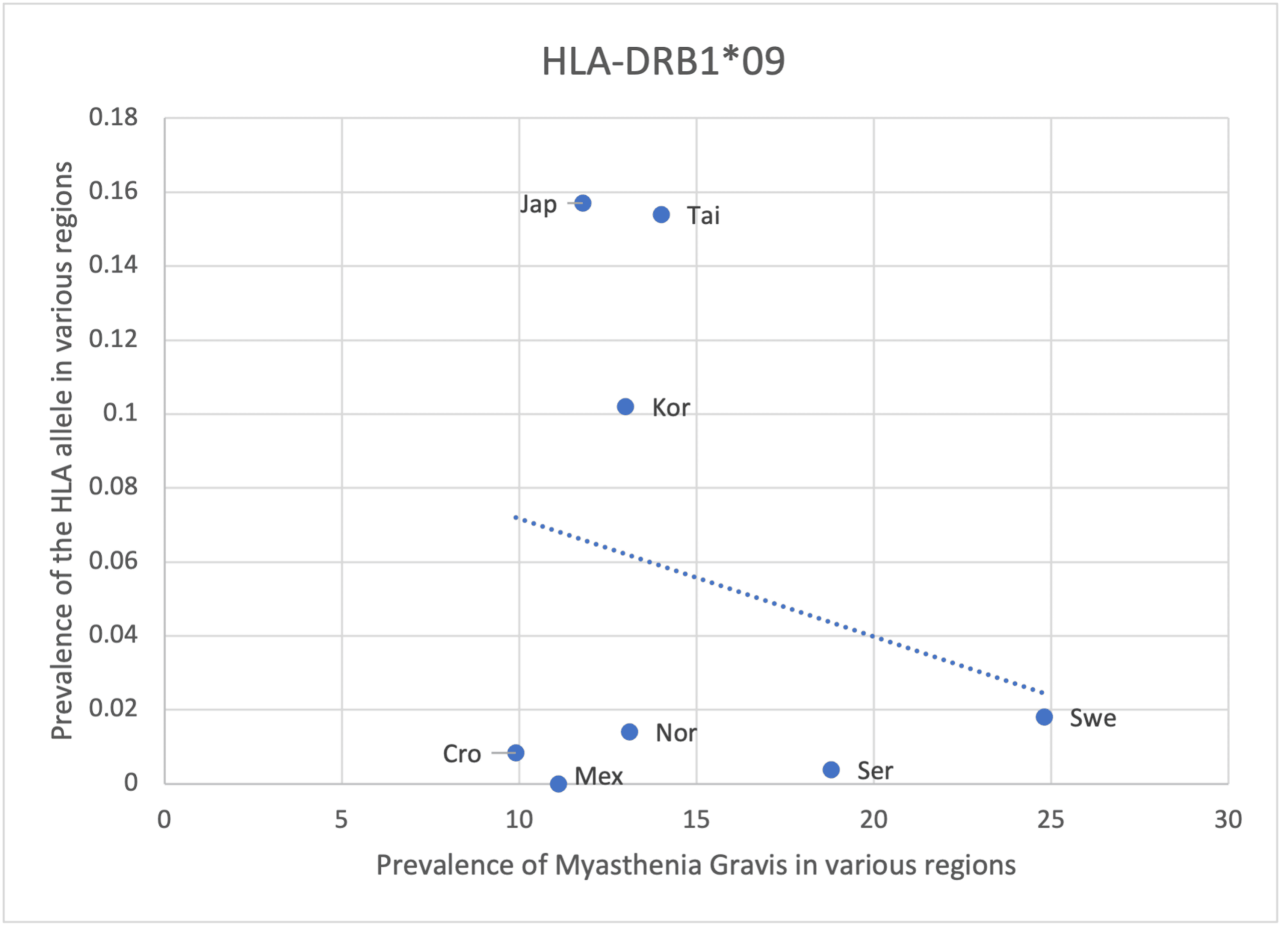
The correlation between the frequency of HLA-DRB1*09 in the various regions studied and the prevalence of MG in those same regions HLA: human leukocyte antigen, MG: myasthenia gravis

**Figure 10:**
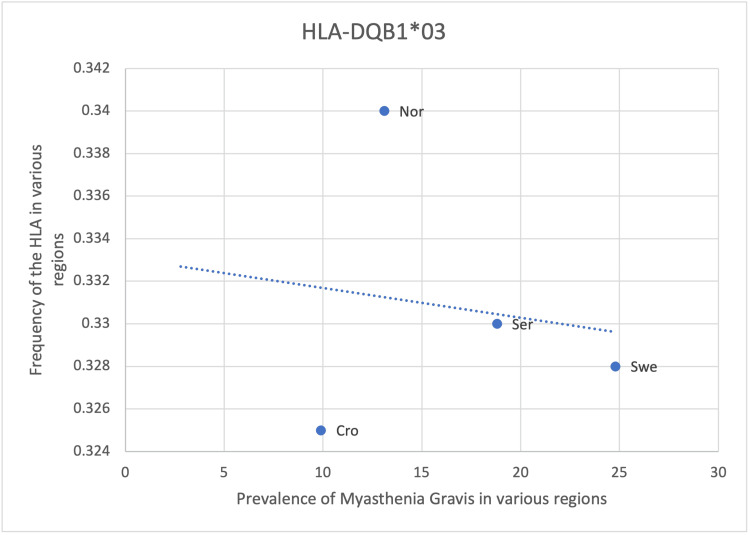
The correlation between the frequency of HLA-DQB1*03 in the various regions studied and the prevalence of MG in those same regions HLA: human leukocyte antigen, MG: myasthenia gravis

Results from HLA-DRB1*09 and DQB1*03 suggest a negative correlation with the prevalence of MG, which contradicts the positive association described in Fekih-Mrissa et al. [15], Xie et al. [17], Fernández-Mestre et al. [18] (Table 8, Figure 9, Figure 10). The prevalence of DRB1*09 was higher in Asian countries as compared to European and American countries (Figure 9). Due to the limited availability of data, only European countries are represented in HLA-DQB1*03 (Figure 10). Therefore, comparisons in HLA frequency between regions are not possible. Statistical analysis also revealed the correlation to be insignificant with a p-value of > 0.05 (Table 8). Incidental findings or other factors mentioned previously could cause a decrease in the frequency of these alleles and the prevalence of MG (Table 8, Figure 9, Figure 10).

Discussion

Limitations of the Research

Papers analysing the prevalence of MG were restricted to European, Asian and American countries and lacking in other areas of the world such as Africa. This limited the database available to fully analyse the correlation of selected HLA alleles to MG prevalence around the world. 

Similarly, when investigating the prevalence of HLA alleles using the website allelefrequencies.net, certain regions where there was a prevalence of MG had no correlating allele frequency in the database. This limited the data available and meant that each of the HLA alleles had different countries represented in the result.

The rate of MG is increasing with an ageing population. The prevalence of MG is obtained from only the latest available research in the literature. Therefore, it may not be representative of the current prevalence of MG in these populations.

Critical Analysis of Research Papers 

Critical analysis of papers associating HLA alleles with MG: The HLA alleles found to be associated with MG were already well-established. As such, critical analysis of the papers associating HLA alleles with MG assumed the papers to be robust and reliable.

Critical analysis of papers investigating the prevalence of MG in various regions: Critical analysis of the papers revealed similarities and differences in the sample size, methodology and diagnostic criteria for MG. 

Sample size is a good indication of the reliability of the data and the representation of the data on the whole population. The methodology for obtaining the sample size differed between certain papers. All papers extracted MG prevalence data from medical institutions. Lee et al. [19] and Lai et al. [20] used the National Health Insurance (NHI), which provided the most comprehensive database for the prevalence of MG for the whole population (Table 6). Anderson et al. [25] and Fang et al. [27], based part of the prevalence of MG on the pharmacy registry while Anderson et al. [25] also included an additional nationwide database (Table 6). Hospital databases were the source in Murai et al. [21], Eaton et al. [22], Lavrnic et al. [24] and Tolosa-Tort et al. [26], and this only included the patients who visited the hospital (Table 6). As such, those who have the condition without a hospital diagnosis were excluded. Although Zivadinov et al. was also hospital-based, it covered a wider variety of healthcare, including outpatient, inpatient, primary care, death certificates etc and is therefore more comprehensive for MG prevalence (Table 6) [23]. Heidi et al. aimed to establish the first nationwide registry and prevalence was based on specialist care (Table 6) [28]. However, due to the rarity of the disease and hence the lack of experience of doctors, underdiagnoses was a likely reason for the small sample size observed. Underdiagnosis was also an issue in Lee et al. [19], especially of the elderly population and in Eaton et al. [22], who only used records for those diagnosed with MG after 1977 when the registry began (Table 6).

The populations covered in the prevalence vary between papers, thus having some impact on the data provided. Data from Heidi et al. was mainly obtained from affluent areas and areas of poverty were under-registered due to a lack of healthcare access (Table 6) [28]. This diminishes the cross-sectional representation of the data of the whole population. Similarly, Tolosa-Tort et al. [26] used data mainly obtained from bigger cities (Table 6). All other papers covered the population across the regions more or less uniformly (Table 6).

Diagnostic criteria of MG were more comprehensive in certain papers. The criteria for diagnosis were based on various methods. Lee et al. [19] and Lai et al. [20] used diagnostic coding to obtain cases (Table 6). The diagnosis by Lee et al. was done by specialist doctors including neurologists, paediatricians and ophthalmologists, thus giving reliability to the data from coding (Table 6) [19]. For Lai et al., the reliability of the diagnosis was based on specific MG tests including an assay of acetylcholine receptor antibodies (AChR-Ab) and single-fibre electromyography in addition to coding (Table 6) [20]. Murai et al. [21] and Zivadinov et al. [23] similarly stated the diagnosis was based on specific tests for MG but also includes symptoms and signs, which provides a more variable diagnostic criterion (Table 6). Pharmacological data to record the prescription of pyridostigmine, which is only used for MG, was used as diagnostic criteria for Andersen et al. [25] and Fang et al. [27] (Table 6). Fang et al., however, also included inpatients and hospital-based specialist outpatients as part of the diagnostic criteria (Table 6) [27]. Heidi et al. studied a wide variety of autoimmune diseases and therefore specific criteria for MG diagnosis were not mentioned (Table 6) [28]. Likewise, Tolosa-Tort et al. did not comment on the specific diagnostic criteria (Table 6) [26]. Overall, the diagnostic criteria for MG were not transparent, except in Lai et al., Murai et al. and Zivadinov et al. (Table 6) [20,21,23]. 

Papers were limited to 2017 to purpose future research into the effects of SARS-CoV-2 (COVID-19) on the prevalence of MG. A systematic review of primary studies of every design from January 2000 to October 2021 showed an increased risk of developing new-onset MG for patients infected with COVID-19 [29]. Likewise, the risk of a myasthenic crisis, respiratory failure and even mortality was noted to be higher in these patients [29]. The mechanism of action is thought to be due to a SARS-CoV-2 infection causing the release of inflammatory cytokines and a cytokine storm [29]. This results in immune system dysregulation and hence increased risk of developing MG in susceptible patients [29,30].

Similarly, a case-reports-based review of 14 publications with 18 cases suggested a relationship between COVID-19 and new-onset MG [31]. However, this review concluded that direct causality between COVID-19 and MG could only be established by reviewing the epidemiological changes in the prevalence of MG post the COVID-19 pandemic [31].

The impact of SARS-CoV-2 on the prevalence of MG in various geographical locations around the world would be interesting to study. Furthermore, whether this impact has any bearing on the association between the established HLA alleles and MG in this study can be revisited. As such, a repeat review of the correlation between the prevalence of MG and the frequency of class II HLA alleles in various geographical locations around the world after the COVID-19 pandemic can be warranted.

## Conclusions

The study intended to identify HLA class II alleles that are positively associated with the prevalence of myasthenia gravis and the correlation between the regional variation of these alleles and the prevalence of MG. Analysis of the results revealed two HLA alleles, HLA DRB1*04:04 and HLA-DRB1*03, to be significantly positively associated with MG prevalence. Identifying these alleles associated with MG can provide an immunological link for the development of the disease. Possible screening tools can be implemented to screen for these susceptible HLA alleles in patients at risk of developing MG. This may aid in diagnosis and hence provide earlier intervention for the disease. Patterns of regional variation were also observed in European countries, especially northern Europe, having a higher prevalence of these alleles as compared to other regions investigated. By contrast, HLA-DRB1*01, DRB1*04, DRB1*15:01, DRB1*09 DQB1*02 and DQB1*03 did not have a significant correlation with MG prevalence. As such, the research cannot conclusively state the association of these alleles with MG.

To further the research, class I and atypical HLA alleles could be searched for their association with MG prevalence. Moreover, HLA alleles associated with different sub-types of MG can be explored. This may help potentiate a screening tool to identify the sub-type of MG in patients who have already been diagnosed with the disease. In Type 1 diabetes, initial associations with HLA-DR alleles were later found to be stronger to HLA-DQ alleles often inherited in the same haplotype. It would be of interest to explore particular HLA-DQ alleles commonly occurring in haplotypes with HLA DRB1*04:04 and HLA-DRB1*03. Lastly, as previously mentioned, the impact of the COVID-19 pandemic could give rise to epidemiological changes in the prevalence of MG. Further research on the association between HLA class II alleles and MG post the COVID-19 pandemic in various geographical locations around the world could reveal a unique impact of the pandemic on these subsets of patients.
